# Modeling the *Colchicum autumnale* Tubulin and a Comparison of Its Interaction with Colchicine to Human Tubulin

**DOI:** 10.3390/ijms18081676

**Published:** 2017-08-02

**Authors:** Ivana Spasevska, Ahmed T. Ayoub, Philip Winter, Jordane Preto, Gane K.-S. Wong, Charles Dumontet, Jack A. Tuszynski

**Affiliations:** 1 INSERM 1052/CNRS 5286/UCBL, Cancer Center Research of Lyon, 69008 Lyon, France; ivana.spasevska@univ-lyon1.fr (I.S.); charles.dumontet@chu-lyon.fr (C.D.); 2 Medicinal Chemistry Department, Heliopolis University, Cairo-Belbeis Desert Rd, El-Nahda, El Salam, Cairo Governorate 11777, Egypt; atayoub@ualberta.ca; 3 Department of Oncology, University of Alberta, Edmonton, AB T6G 1Z2, Canada; pwinter@ualberta.ca (P.W.); preto@ualberta.ca (J.P.); 4 Department of Biological Sciences and Department of Medicine, University of Alberta, Edmonton, AB T6G 2E9, Canada; gane@ualberta.ca

**Keywords:** tubulin, colchicine, *C. autumnale*, binding site, cytotoxicity, molecular modeling

## Abstract

Tubulin is the target for many small-molecule natural compounds, which alter microtubules dynamics, and lead to cell cycle arrest and apoptosis. One of these compounds is colchicine, a plant alkaloid produced by *Colchicum autumnale*. While *C. autumnale* produces a potent cytotoxin, colchicine, and expresses its target protein, it is immune to colchicine’s cytotoxic action and the mechanism of this resistance is hitherto unknown. In the present paper, the molecular mechanisms responsible for colchicine resistance in *C. autumnale* are investigated and compared to human tubulin. To this end, homology models for *C. autumnale* α-β tubulin heterodimer are created and molecular dynamics (MD) simulations together with molecular mechanics Poisson–Boltzmann calculations (MM/PBSA) are performed to determine colchicine’s binding affinity for tubulin. Using our molecular approach, it is shown that the colchicine-binding site in *C. autumnale* tubulin contains a small number of amino acid substitutions compared to human tubulin. However, these substitutions induce significant reduction in the binding affinity for tubulin, and subsequently fewer conformational changes in its structure result. It is suggested that such small conformational changes are insufficient to profoundly disrupt microtubule dynamics, which explains the high resistance to colchicine by *C. autumnale*.

## 1. Introduction

Microtubules (MT), the main components of the cytoskeleton, are formed of polymers of α and β tubulin expressed as stable heterodimers. They organize to create a stable interphase microtubule network, and the highly dynamic mitotic spindle, during cell division. In eukaryotic cells, microtubules play crucial roles in a number of cellular functions, including cell signaling, cellular transport, morphogenesis, cell motility, and cell division [1]. Any alteration in tubulin polymerization disrupts the cell homeostasis and leads to mitotic arrest, eventually resulting in cell death [2]. Several natural compounds of various structures, such as colchicine, vinca alkaloids and taxanes have been found to bind to tubulin and alter MT dynamics. Hence, these compounds have demonstrated an immense potential in clinic as anti-mitotic drugs [3]. Whereas vinca alkaloids and taxanes turned out to be effective chemotherapeutic agents and have found their way into anti-cancer treatments, colchicine and its analogues and derivatives are still in preclinical stages of development due to their high toxicity and severe side effects.

**Figure 1:**
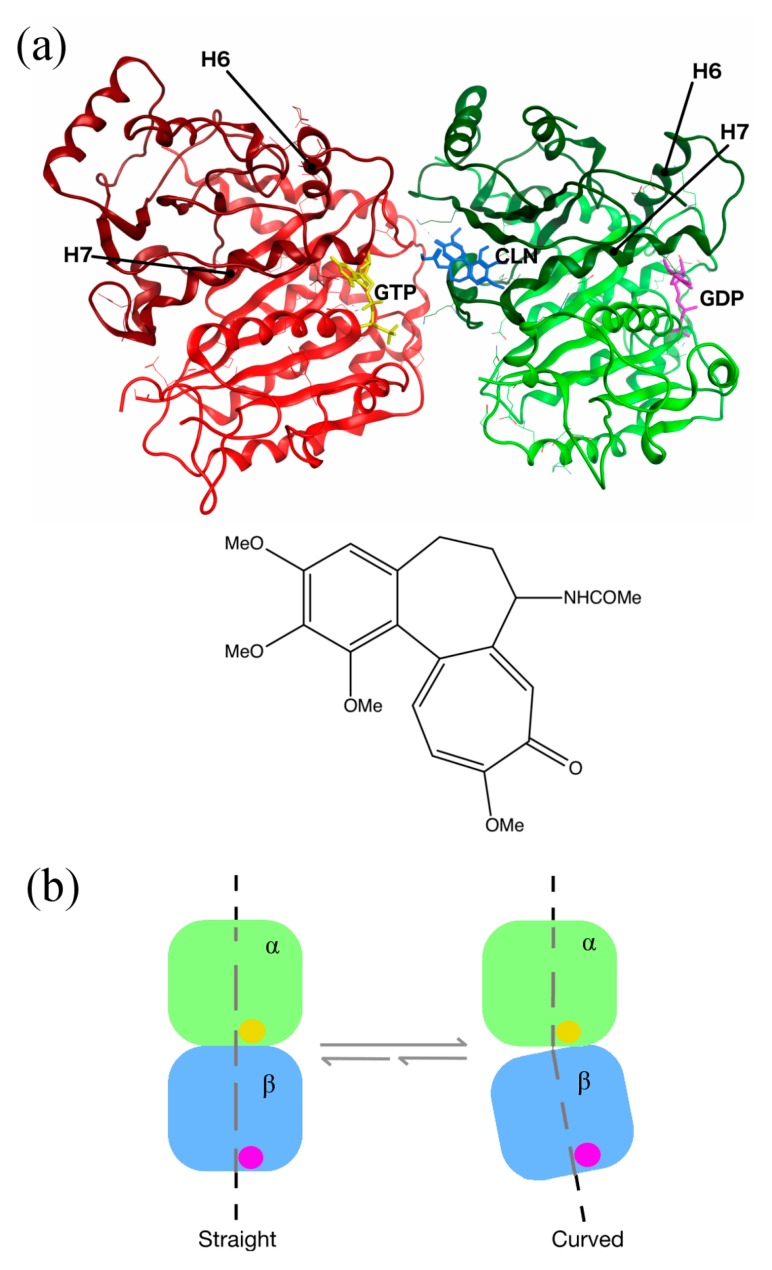
Structure of human tubulin heterodimer bound to colchicine, guanosine triphosphate (GTP) and guanosine diphosphate (GDP). (**a**) Location of the colchicine-binding site: α and β tubulins are presented in red and green ribbon structures, respectively, with the intermediate domains in darker colors. GTP and GDP are shown in yellow and purple, respectively. Colchicine (CLN) is represented in blue, with a zoom on its chemical structure on the picture below (the image was prepared with MOE2012.10 [11], adapted from the Protein Data Bank (PDB) ID:1SA0). (**b**) Schematic representation of the conformational changes in tubulin, undergoing from straight to curved structures. The α subunit is bound to GTP (yellow ball), and the β subunit to GDP (purple ball). The tubulin dimer representation was redrawn based on the information obtained from Ravelli et al.’s 2004 study [6].

Colchicine is a plant alkaloid that was first isolated in 1820 from the leaves of *Colchicum autumnale* (autumn crocus, also known as meadow saffron). Since then, colchicine has been used—and continues to be used—for the treatment of gout, familial Mediterranean fever, and pericarditis [4]. Although it has been used as a therapeutic agent for many years, colchicine’s biological action remained unknown until 1968, when Taylor et al. recognized tubulin as its biological target [5]. In 2004, Ravelli et al. [6] identified a colchicine-binding site at the interface of the α-β tubulin heterodimer (Figure 1a). While the binding site for colchicine is located between the α and β tubulin monomers, the principal interaction zone is located on the β subunit. The colchicine-binding pocket was identified within the intermediate domain of β tubulin. Three distinct regions of interaction, which include several amino acids, can be identified within a 6 Å cutoff range of the bound colchicine [7,8], namely, residues 235–260, residues 310–320, and residues 350–360. The binding of colchicine to human tubulin induces important structural changes within the tubulin subunits, shifting from a straight to a curved conformation that renders the tubulin dimer assembly incompetent for microtubules. Taking the N-terminal domain of β tubulin as a reference, in going from a straight to a curved conformation, the following conformational changes occur: (I) the H7 helix of the β subunit translates, along with an important rotation of the intermediate domain; (II) the H6–H7 loop and the H6 helix protrude at the longitudinal interface between tubulin subunits [9] (Figure 1). The presence of tubulin dimers with curved conformations leads to the blocking of microtubules polymerization, which induces their depolymerization. The MT depolymerization triggered by the tubulin–colchicine complex is the main cause for its cytotoxicity.

In a previous paper [10], some of the present authors analyzed the structure of tubulin expressed in the yew tree, and compared it to that expressed in *Homo Sapiens*. It was concluded that the yew tree tubulin has an overall structural similarity compared to human tubulin, but there is a very significant difference in the paclitaxel-binding site. Consequently, the toxin produced by the yew tree, paclitaxel, has a much lower binding affinity for yew tree tubulin than human tubulin. In humans, paclitaxel is a cytotoxic agent used in cancer chemotherapy, but the yew tree is insensitive to it. In the present paper, we have attempted to analyze a similar situation for colchicine and the plants that produce it. Although colchicine has a high toxic effect on human cells, it does not affect its source plant. Produced as a secondary metabolite of *C. autumnale* and *Gloriosa superba*, colchicine is involved in these plants’ self-defense mechanisms against pathogens. Interestingly, both plants and humans express the target protein for the action of the toxin. However, only the human cells are susceptible to it. As tubulin is a highly conserved protein in eukaryotic cells, we explored the reasons for the resistance of *C. autumnale* to its own toxin given the fact that, at the same time, human cells are extremely sensitive to colchicine. It was suggested that colchicine binding to *C. autumnale* tubulin either does not induce conformational changes, or induces less of a conformational change. This is unlike the binding of colchicine to human tubulin, which results in an important conformational shift in the tubulin structure, that is, a shift from a straight conformation to a curved one. In order to test out this hypothesis, molecular models for both human and *C. autumnale* tubulins were created. For each model, colchicine-binding affinity was estimated, and possible conformational modifications due to the binding were investigated.

This study summarizes the interaction of the β tubulin of *C. autumnale* with colchicine, as assessed by comparative genomics, computational studies, and molecular modeling. Comparison of the molecular model for the *C. autumnale* tubulin dimer with the human model showed that the resistance to colchicine is related to amino acid substitutions, leading to a weaker interaction with colchicine. Contrary to human tubulin, it is suggested that conformational changes associated with the binding of colchicine to *C. autumnale* tubulin do not significantly disrupt MT dynamics, thus explaining the high resistance of colchicine for *C. autumnale.*

## 2. Results

### 2.1. Colchicum autumnale and Human Tubulin Sequences

Plant tubulin protein sequences were assembled from the 1000 Plants Initiative (1KP) transcriptome database [12]. Two different species of colchicine-producing plants were available: *C. autumnale* and *G. superba*. After quality trimming of the sequences, only one α tubulin sequence was available: sample SFCT from *C. autumnale*. We decided to use a sequence for β tubulin from the same plant. Instead of using consensus sequences assembled from all available sequences, we chose the NHIX sample sequence, since it is the only one obtained from the plant bulb, and it is known that plant bulbs produce colchicine. Moreover, no significant differences between the available β sequences were observed (more then 94% identity and 97% similarity).

As for the human tubulin sequences, we selected the tubulin α chain (TUBA1C) (UniProt AC: Q9BQE3), since it has the highest expression signals averaged over all samples; and the tubulin βI isoform (TUBB) (UniProt AC: P07437), since it is an isoform ubiquitously expressed, and it is the most abundant isotype in most tumors.

**Figure 2:**
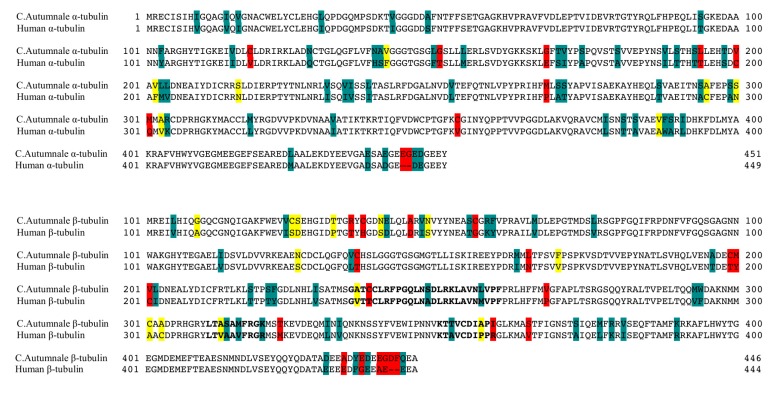
Sequence alignment of the *Colchicum autumnale* tubulin with human tubulin. The *C. autumnale* α tubulin sequence (1KP SFCT) was aligned to the human α chain tubulin (TUBA1C) (PDB ID: Q9BQE3), and the *C. autumnale* β tubulin sequence (1KP: NHIX) was aligned to the human tubulin βI isoform (TUBB) (PDB ID: P07437). The sequence alignment was performed using ClustalX 2.0 [14]. Identical residues are shown in a white background. “Strongly” conserved residues within the same group are shown in cyan, and “weakly” conserved residues (within the same group, but different charge/structure) are shown in yellow. Residue substitutions are shown in red. The residues identified in the colchicine-binding domain are represented with bold letters.

In order to compare the amino acid sequences of human tubulin and those obtained for the *C. autumnale* tubulin, we performed a sequence alignment using the Basic Local Alignment Search Tools (BLAST) webserver [13]. The multiple sequence alignment was performed between the human α and β tubulin isoforms TUBA1C and TUBB, and the selected sequences for *C. autumnale* α and β tubulins. We analyzed the primary structures for the full-length proteins, as well as for the colchicine-binding domain. Our results showed 86% sequence identity for all comparisons, and sequence similarities were found to be 93% and 96% for α and β tubulins, respectively. The comparison of the primary structure of the colchicine-binding domains in both human and *C. autumnale* tubulins also revealed a high percentage of identity (86%) and similarity (94%) (Figure 2). Interestingly, the several amino acids that differ in the colchicine-binding domain were common for other plants, suggesting that the difference in the amino acid sequences is not crucial for the differential colchicine binding to human and *C. autumnale*, but is a characteristic of the evolutionary trend. Phylogenetic trees for both α and β tubulins, including plant and animal groups, are provided in the supporting information (Figures S1 and S2).

### 2.2. Homology Models

**Figure 3:**
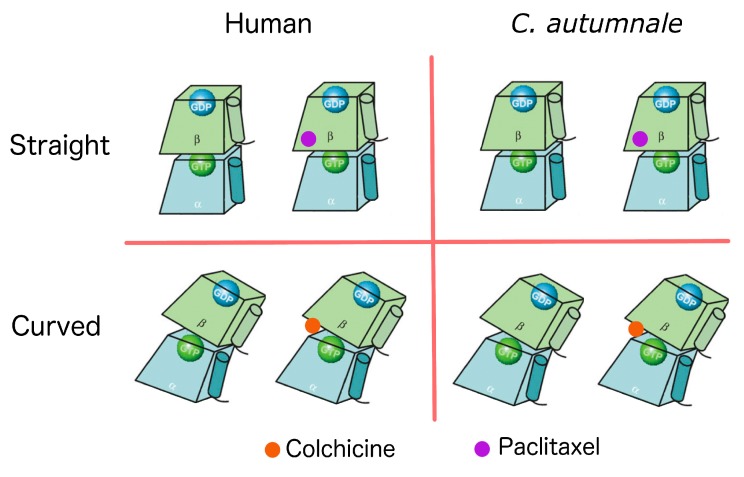
Schematic representation of each of the eight homology models constructed for this study. The α tubulin is represented in blue, and the β tubulin in green. Each subunit is bound to a nucleotide. The α subunit is bound to GTP (green ball), and the β subunit to GDP (blue ball). Colchicine is represented in orange, and paclitaxel in purple. The graphical representation of the tubulin dimers was generated based on the information obtained from Krebs et al.’s 2005 study [16].

**Figure 4:**
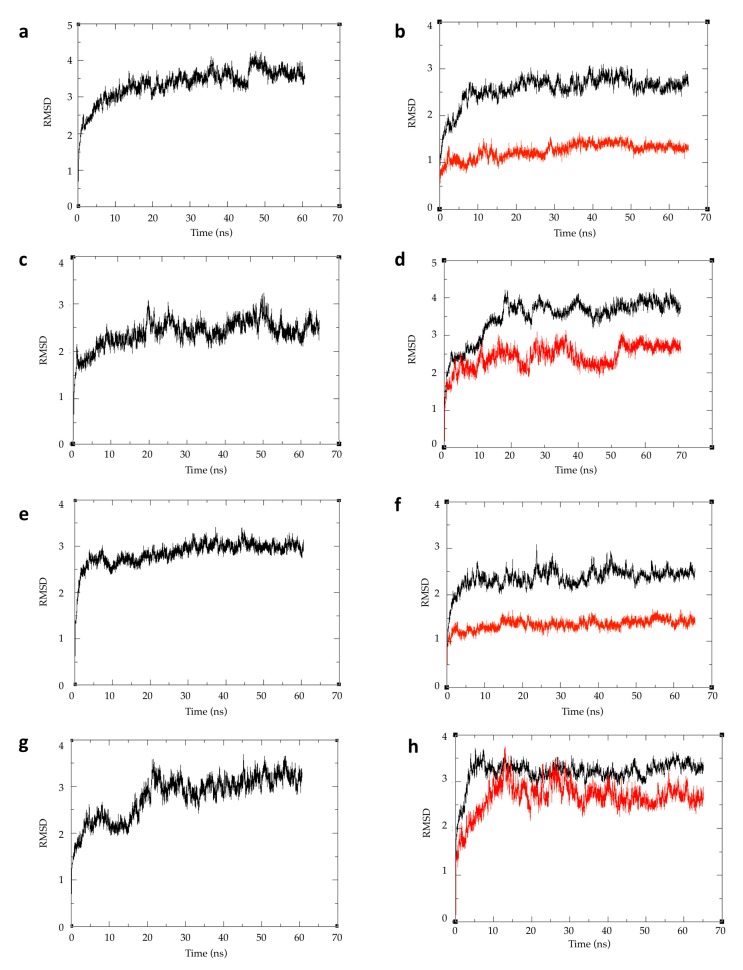
The root-mean-square deviation (RMSD) from the initial minimized structures for each of the studied systems. Plot (**a**) is human tubulin in straight conformation without ligand, plot (**b**) is human tubulin in straight conformation bound to paclitaxel, plot (**c**) is human tubulin in curved conformation without ligand, plot (**d**) is human tubulin in curved conformation bound to colchicine. Plot (**e**) is *C. autumnale* tubulin in straight conformation without ligand, plot (**f**) is *C. autumnale* tubulin in straight conformation bound to paclitaxel, plot (**g**) is *C. autumnale* tubulin in curved conformation without ligand, plot (**h**) is *C. autumnale* tubulin in curved conformation bound to colchicine. RMSD analyses are shown for both full-length protein (black curved) and the ligand-binding domain when the ligand is present (red curve).

To examine the differences between human and *C. autumnale* tubulins, and to test our hypothesis that the *C. autumnale* tubulin is resistant to colchicine, we performed molecular modeling of a tubulin heterodimer for both species. In the absence of a *C. autumnale* X-ray resolved crystal structure of tubulin, we constructed homology models using the following crystal structures available from the Protein Data Bank (PDB): 1JFF, a refined structure of the α-β tubulin from zinc-induced sheets stabilized with paclitaxel (the “straight” conformation of tubulin) and obtained from bovine [15], and 1SA0, a tubulin–colchicine and stathmin-like domain complex (the “curved” conformation of tubulin) obtained from bovine and rat [6]. Both templates were used for threading of the *C. autumnale* genes and human genes for the α and β tubulins. The generated homology models were expected to have a similar resolution to the original structures i.e., around 3.5 Å (3.50 Å for 1JFF, and 3.58 Å for 1SA0). The sequence alignment of the *C. autumnale* and human tubulin genes to the 1JFF and 1SA0 templates indicated high sequence homology: 81% sequence identity for *C. autumnale*, and 97% for the human sequences. In order to calculate the colchicine-binding energies to human and *C. autumnale* tubulins, respectively, as well as to visualize any conformational changes, molecular modeling was conducted on the holo (ligand bound) and apo (no ligand bound) forms of the protein. At the same time, molecular modeling was performed using paclitaxel as a ligand (Figure 3). Structures of colchicine, paclitaxel and other colchicine-related compounds are provided in the supporting information (Figure S3). The binding of paclitaxel to human tubulin has already been studied, and it is known that paclitaxel binding to β-tubulin stabilizes the tubulin dimer in the straight conformation. In this study, we used paclitaxel as a positive control for the straight conformation and for the binding free energy calculations. Each homology model was energy minimized to refine the geometry of each system, and to relieve any bad contacts among the added hydrogen atoms. The geometry-optimized systems were simulated in physiological conditions until they reached equilibrium. The root-mean-square deviation (RMSD) of each complex backbone with respect to the initial protein structure was plotted for the full-length protein and the ligand-binding domain (Figure 4). Although the RMSDs of the holo form are higher than those of the apo form, all of the systems reached equilibrium after approximately 50 ns, indicating that the 65 ns simulations are adequate for performing the binding free energy analysis.

### 2.3. Free Energy of Binding Calculations

In order to investigate whether colchicine has a different affinity between human and *C. autumnale* tubulins, we calculated the binding free energies using the Molecular Mechanics Poisson–Boltzmann Surface Area (MM/PBSA) technique [17]. The MM/PBSA method combines molecular mechanics and solvation energies on the molecular dynamics (MD) trajectory. Further estimates of the binding energy performed from another popular continuum solvation model, i.e., the Molecular Mechanics Generalized-Born Surface Area (MM/GBSA) technique were provided (Table 1). The main difference between the MM/PBSA and MM/GBSA techniques lies in the estimation of the polar contribution of the solvation free energy (see Equation (1), Section 4.4). For MM/PBSA, this contribution is typically obtained by solving the Poisson–Boltzmann equation while the generalized born (GB) model is used in the MM/GBSA approach [18].

All binding energy calculations were performed on the MD trajectories during which the complexes were equilibrated, in this case, from 50 to 65 ns.

Our calculations of the binding free energies for paclitaxel have confirmed its binding to human tubulin, with the same affinity found in experimental tests. In addition, our calculations suggested that paclitaxel binds to *C. autumnale* tubulin with almost the same affinity as to human tubulin (Table 1). Although the free energy estimated from the MM/GBSA technique showed a fairly substantial free energy difference, i.e., 3 kcal/mol of paclitaxel had more affinity for human tubulin than for *C. autumnale*, the difference obtained from the MM/PBSA technique is only 1 kcal/mol with an opposite tendency, i.e., paclitaxel binding was slightly more preferential for the *C. autumnale* tubulin. Energy decomposition per residue within 8.0 Å of the bound paclitaxel showed key residues known to interact with paclitaxel (Table 2) [10].

On the other hand, colchicine was clearly found to have high affinity and a preferred binding mode to human tubulin ($\Delta G^{0}$ = −51.79 kcal/mol with the GBSA model and $\Delta G^{0}$ = −76.68 kcal/mol with the PBSA model), but significantly lower affinity to the *C. autumnale* tubulin ($\Delta G^{0}$ = −43.44 kcal/mol with the GBSA model and $\Delta G^{0}$ = −64.71 kcal/mol with the PBSA model) (Table 1). To obtain a better insight into the differences in the binding free energy of colchicine, we performed energy decomposition. We looked into the residues located within an 8.0 Å cut-off range of the bound colchicine (Table 2). In human tubulin, we found eight residues that significantly contribute to colchicine binding. Two residues are located in the α subunit, whereas the other six are located in the colchicine-binding domain in the β subunit. In the case of *C. autumnale*, we found six amino acids that significantly contribute to colchicine binding. Interestingly, five out of the six residues involved in colchicine binding to *C. autumnale* tubulin are also key residues in human tubulin. The exception is βLeu257 in *C. autumnale*, which contributes to colchicine binding, but is not significantly involved in human tubulin binding, although this residue is conserved between the two species. In addition to the common residues in human tubulin, we found that βCys239, βAla248 and βAla314, with βAla248 are the residues with highest energy contributions. Moreover, the contribution of colchicine is different for the two species, with $\Delta G^{0}$ predicted to be −28.22 kcal/mol for human tubulin, and −23.55 kcal/mol for *C. autumnale*.

### 2.4. Atomic Fluctuations

**Figure 5:**
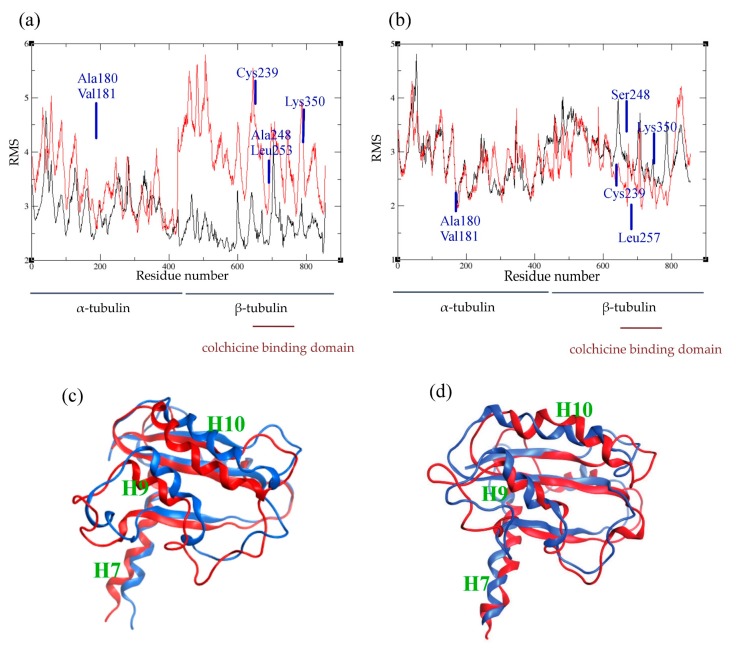
Comparison between the holo and apo forms of tubulin. (**a**,**b**): average atomic fluctuations per residue in human (**a**) and in *C. autumnale* (**b**) tubulins. The tubulin heterodimer is represented in red in the presence of colchicine, and in black in the absence of colchicine. Key amino acids involved in colchicine binding are depicted on the graphs. (**c**,**d**): overlay of the conformations of the colchicine-binding domain of β tubulin in the presence and absence of colchicine. The conformations of the intermediate domain of the human β-tubulin are shown in (**c**) and the ones of the *C. autumnale* β tubulin are depicted in (**d**). The holo form of tubulin bound to colchicine is colored in blue, and the apo form in red.

In order to analyze the behavior of the residues and their flexibility, we calculated the atomic fluctuations for each residue in the presence and absence of the ligand, respectively. In human tubulin, the presence of colchicine did not affect the fluctuations of the residues in the α subunit. However, more fluctuations are induced for residues at the interface between α and β monomers, which suggests a conformational change of the dimer (Figure 5a). Generally, residues in the β subunit show larger fluctuations, except for the residues involved in colchicine binding. These residues are stabilized by the presence of colchicine, confirming their implication in the binding. On the other hand, in *C. autumnale* tubulin, the bound colchicine did not cause any major modifications in the atomic fluctuations (Figure 5b). We observed an increase of atomic fluctuations in the N-terminal tail in the α subunit, but not as large as in human tubulin. In the β subunit, residues are in general more stabilized. This includes residues involved in colchicine binding, especially βLys350. Taken together, these results suggest an important conformational change in human tubulin into a curved shape. This change created a gap between the two subunits, followed by an increasing size of atomic fluctuations at the α–β interface. However, no such significant increase was observed in plant tubulin, which suggests less important conformational changes in that case.

### 2.5. Conformational Changes

In order to examine how the presence of colchicine influences tubulin conformation, we extracted the lowest energy structures obtained from MD simulations and superimposed the structures by aligning the N-terminals of the β tubulin. To investigate the conformational changes, we looked into the β subunit intermediate domain, which is known to be affected by the colchicine binding. By superimposing the human tubulin bound to colchicine with the human tubulin in straight conformation that is unbound to colchicine, we observed a significant dislocation in the intermediate domain, especially in regions involving the helices H7, H9 and H10 (Figure 5c). In *C. autumnale* tubulin, we also observed a shift in the intermediate domain, again, in regions including the H7, H9 and H10 helices (Figure 5d). However, this shift was less important than in human tubulin, confirming that colchicine bound to *C. autumnale* induces less notable conformational changes.

## 3. Discussion

Colchicine, a plant alkaloid, has an enormous pharmacological importance due to its ability to bind to tubulin and inhibit microtubule polymerization, which leads to mitotic arrest. Yet, the immense pharmacological potential of colchicine remains unfulfilled due to its severe side effects. Interestingly, while colchicine is extremely toxic for human cells, the plants that endogenously express colchicine, such as *C. autumnale* and *G. superba*, seem to be unaffected by its cytotoxicity even though they express tubulin, the biological target of colchicine. This observation suggests that there could be a method of redesigning colchicine analogues to enable differential binding between tubulin isotypes in the human body. This might allow for a reduction of side effects, while maintaining efficacy. In this paper, we performed a computational study in order to explore what might be the cause for the resistance of *C. autumnale* to colchicine. For this purpose, molecular modeling of *C. autumnale* tubulin was performed based on the transcriptomic data obtained from the Alberta 1000 Plants Initiative (1KP) database.

Tubulin is a highly conserved protein in all eukaryotic cells, although multiple tubulin isotypes are widespread among eukaryotes. In order to investigate whether the differential colchicine binding to human and *C. autumnale* tubulin is caused by sequence variations, we performed a sequence alignment. The results revealed a high conservation of the amino acid residues with 86% sequence identity between the *C. Autumnale* and the human tubulins. In addition, we compared the colchicine-binding domains in both species. Again, high sequence homology was found. The five residues differing in the colchicine-binding site between human and *C. autumnale* were T238C, A248S, M257L, V258I, and R359I of the β subunit. Interestingly, all of these residues were found to be common among other plants such as *Taxus baccata*, *Podophyllum peltatum*, *Larrea tridentate* and *Catharanthus roseus*. The high conservation of these residues in other plant species suggests that the differential colchicine binding to human and *C. autumnale* tubulin cannot be explained only by sequence variations and substitutions in the colchicine-binding site; it also requires an analysis of evolutionary trends in general.

Since sequence variation is not sufficient to explain colchicine resistance within *C. autumnale*, we investigated whether any difference in the colchicine-binding affinity could be observed between human and *C. autumnale* tubulins. For that purpose, we performed MM/PBSA and compared the binding free energies of both human and *C. autumnale* tubulins. It was found that colchicine has a significantly higher affinity for human tubulin than *C. autumnale* tubulin, even though our results indicated that colchicine is still able to bind to *C. autumnale*. The energy decomposition per residue revealed eight residues that are critical for colchicine binding to human tubulin, with αAla180, βLeu246, and βLeu253 having the highest energy contribution. αAla180 and αVal181 are both hydrophobic amino acids, which establish van der Waals contacts with the methoxy tropone ring (C ring) of colchicine. βCys239, βLeu246, βAla248, βLeu253, βAla314, βLys350 delimit a hydrophobic pocket in tubulin, and interact with the trimethoxy benzene ring (A ring). The thiol group of βCys239 is involved in a hydrogen bond with the methoxy group of the A ring. On the other hand, in *C. autumnale*, fewer residues were involved in the binding of colchicine, with a major contribution of βLeu246 and βLys350. Moreover, βLeu257 was found to be involved in the colchicine binding to *C. autumnale*, but not to human tubulin. Moreover, position 180 in *C. autumnale* is associated with serine, while it is associated with alanine in human tubulin. However, this substitution does not seem to modify colchicine binding, since both amino acids establish van der Waals contacts with the same strength. Another variation occurs at position 248. In human tubulin, βAla248 turns out to be a key residue for colchicine binding, whereas βSer248 in *C. autumnale* tubulin does not seem to be involved. These amino acids are located in the hydrophobic pocket of β tubulin. Alanine, being a hydrophobic amino acid, contributes to hydrophobic interactions with the A ring of colchicine. On the contrary, serine, as a polar amino acid with a hydroxyl group, causes local destabilization in the hydrophobic pocket. Surprisingly, βCys239 was not involved in *C. autumnale*, which suggests a less stable interaction between the A ring of colchicine and β tubulin.

In order to estimate the flexibility of tubulin residues in the presence or absence of colchicine, we looked into the atomic fluctuations of each residue. When analyzing the human tubulin fluctuations, the presence of colchicine induces a higher flexibility of the residues of the β subunit, which are located at its interface within the α subunit. This result confirms the conformational change in human tubulin caused by the binding of colchicine. On the contrary, no significant changes in the residues’ flexibility were observed in *C. autumnale*, both in the presence and absence of colchicine. This indicates that *C. autumnale* tubulin is more rigid than human tubulin when bound to colchicine, and also undergoes fewer conformational changes. These results were also confirmed by the superposition of the structures bound and unbound to colchicine. The minor shift in the intermediate domain of *C. autumnale* β tubulin indicates slight changes in tubulin conformation. Taken together, these findings indicate that colchicine is able to bind to tubulin in *C. autumnale* in the identified colchicine-binding domain, but with a weaker affinity when compared to human tubulin. This low affinity is mostly due to weak interactions involving the A ring of colchicine, which is known to be a critical pharmacophore in the binding to tubulin. Analogues and derivatives having the same A ring as colchicine, such as podophyllotoxin and cornigerine, have proven the crucial role of the A ring in this interaction [19]. Weak interactions of the A ring in *C. autumnale* contribute to a decrease in the total binding energy of colchicine, thus resulting in fewer conformational changes in the tubulin structure. These slight conformational changes can be viewed as the result of forming reversible pre-equilibrium complexes without evolving to a stable tubulin–colchicine complex [20]. Moreover, these conformational changes seem not to alter the microtubules polymerization and their dynamics. In order to further investigate the different conformational changes in tubulin induced by colchicine binding, principal component analysis (PCA) can be performed in the future [21]. However, this is outside the scope of the present study.

An experimental assay on binding affinity would be necessary to confirm the binding free energy calculations. Since βSer248 seems to cause destabilization in the hydrophobic binding pocket, experiments with βAla248 mutated to βSer248 in human tubulin might give a better understanding of the binding site and its interaction with the A ring. It may also be interesting to investigate whether βSer248 in *C. autumnale* tubulin includes any post-translational modifications, such as phosphorylation.

In conclusion, this study reported on the features of the *C. autumnale* tubulin protein and its colchicine-binding site. It provides a better understanding of the interaction of colchicine and its target tubulin in plants endogenously expressing colchicine, and broadens our understanding of this pharmacologically important drug–protein interaction. Since colchicine-binding site agents are examples of tubulin-binding agents, which have not yet reached clinical applications in cancer treatment, it is crucial to understand the biological relevance of the colchicine-binding site. Colchicine has an immense but as yet unrealized potential as an antimitotic drug. The dozens of colchicine derivatives and analogues under development should soon result in clinical trials leading to novel anti-cancer therapeutics. Understanding what types of differences provide resistance against this toxin can aid in the understanding of drug resistance in human cancers as well as contribute to the creation of more specific and more efficacious drugs that bind to the same binding site as colchicine. In a future publication, we intend to examine a similar relation between the tubulin structures expressed by all remaining plants that produce toxins, and the binding affinity for these toxins. We intend this future study to validate the generalization of the hypothesis posed in the present study, that the binding sites are suitably modified to lower the affinity for its own toxic agents. We have seen this in the case of colchicine and *C. autumnale* and *G. superba*, as well as in the case of *paclitaxel* and the yew tree.

## 4. Materials and Methods

### 4.1. Sequence Assembly

Plant tubulin protein sequences were assembled from the 1000 Plants Initiative (1KP) transcriptome database [12] using the following method. Sequence read data were downloaded in FASTQ format from the 1KP data server for the two plant species producing colchicine: *C. autumnale* and *G. superba*. There were three samples available for *C. autumnale*, and only one sample available for *G. superba*. The FASTQ files were trimmed to remove low-quality reads, using a quality threshold of Q20 with the FASTX-Toolkit (The Apache Software Foundation, Forest Hill, MD, USA).

Human tubulin protein sequences were obtained from UniProt [22]. Tubulin α chain (TUBA1C) (Q9BQE3) was chosen as reference sequence for human α tubulin, and βI isoform (TUBB) (P07437) was chosen for human β tubulin. Sequence analysis was performed using BLAST [13], and multiple sequence alignment was performed with ClustalX 2.0 software (UCD Conway Institute, Dublin, Ireland) [14].

### 4.2. Homology Modeling

As mentioned before, homology models were constructed using 1JFF and 1SA0 templates for the straight and the curved conformations of tubulin, respectively. Coordinates of missing residues within the PDB crystal structure 1JFF were obtained from the 1TUB structure of tubulin after RMSD alignment of the two structures [23].

*C. autumnale* α tubulin (1KP sample code SFCT) and β tubulin (1KP sample code NHIX) sequences, as well as human α tubulin (Q9BQE3) and β tubulin (P07437) sequences, were aligned to both 1JFF and 1SA0 structures using the MOE 2012.10 software (Montreal, QC, Canada) [11]. For each target sequence, the maximum number of independent models constructed, available via the “maximum of mainchain models” option in MOE, was set to five. Final models were chosen based on GA431, DOPE and molpdf scores, as well as the lowest RMSD alignment with the template structure. Eight homology models were created in total, four for the plant tubulin dimer, and four for the human tubulin dimer.

The missing hydrogen atoms were added using the tLEAP module of AMBER 12 (San Francisco, CA, USA) [24] with the AMBER12SB force field. Each protein model was solvated in 12 Å box of TIP3P water and neutralized with 30 atoms of Na^+^. In order to bring the salt concentration to the physiological value of 0.1 M, 64 atoms of Na^+^ and Cl^−^ were added.

Protonation states of all ionizable residues were calculated at pH 7 using the program PROPKA 3.1 (GitHub, San Francisco, CA, USA) [25].

The homology models were then energy minimized with periodic boundaries using the AMBER 12 software (San Francisco, CA, USA) [24]. A first minimization run was performed for 2000 cycles with a cutoff of 8.0 Å and heavy restraints on all backbone atoms. A second minimization run was performed for 4500 cycles with a cutoff of 8.0 Å and no restraints.

### 4.3. Molecular Dynamics Simulations

The fully minimized structures were used as starting configurations for the subsequent MD simulations. The MD simulations were carried out in three steps: heating, density, and production. First, each solvated system was heated to 300 K for 50 ps, with weak restraints on all backbone atoms. Next, density equilibration was carried out for 50 ps of constant pressure equilibration at 300 K, with weak restraints. Finally, MD production runs were performed on all systems for 65 ns, during which atomic coordinates were saved every 10 ps. The runs were conducted on 8 Central Processing Units (CPUs) in each case, which enabled the simulation of the protein dynamics at a rate of approximately 0.3–0.4 ns per day with the SANDER module of Amber 12 [24]. Each trajectory produced after a MD simulation was examined. The MD trajectories were analyzed using the ptraj module of AMBER 12 utilities to obtain the RMSD and the total energy. The structures with the lowest energy were extracted using the ptraj module of AMBER 13 tools [24], and superposed with the MOE 2012.10 software (Chemical Computing Group, Montreal, QC, Canada) [11].

### 4.4. Binding Free Energy Calculations

The Molecular Mechanics Poisson–Boltzmann Surface Area (MM/PBSA) technique was used to calculate the free energy associated with ligand binding [17]. This method combines molecular mechanics with continuum solvation models. The total free energy is estimated as the sum of average molecular mechanical gas phase energies ($E_{MM}$) and solvation free energies ($G_{solv}$) of the binding reaction:

(1) $$G=E_{MM}+G_{solv}$$

The total molecular mechanical ($E_{MM}$) energy can be further decomposed into contributions from electrostatic ($E_{ele}$), van der Waals ($E_{vdw}$), and internal energies ($E_{int}$). The molecular mechanical ($E_{MM}$) energy of each snapshot during the last 15 ns of the MD simulation was calculated using the SANDER module of Amber 12 [24], with all pairwise interactions assuming a dielectric constant (ε) of 1.0. The solvation free energy ($G_{solv}$) was estimated as the sum of electrostatic solvation free energy, calculated by the finite-difference solution of the Poisson–Boltzmann equation in the Adaptive Poisson–Boltzmann Solver (APBS) and non-polar solvation free energy, calculated from the solvent-accessible surface area (SASA) algorithm. Applying the thermodynamic cycle for each tubulin-colchicine and tubulin-paclitaxel complex, the binding free energy was approximated by:

(2) $$\Delta G^{0}=\Delta G_{gas}^{Lig-Prot}+\Delta G_{solv}^{Lig-Prot}-\left\{\Delta G_{solv}^{Prot}+\Delta G_{solv}^{Lig}\right\}$$

Here, $\Delta G_{gas}^{Lig-Prot}$ represents the free energy per mole for the non-covalent association of the ligand–protein complex in vacuum (gas phase) at a representative temperature, while $\left(-\Delta G_{solv}\right)$ stands for the work required to transfer a molecule from its solution conformation to the same conformation in vacuum (assuming that the binding conformation of the colchicine protein complex is the same in solution and in vacuum). The decomposition of the binding free energy per residue was also calculated using the MM/PBSA technique [17]. Only the residues within an 8 Å cutoff range of the ligand were taken into account for the calculations. Less than an hour was typically needed to estimate binding energies on 8 CPUs, each using the MMPBSA/MMGBSA technique.

### 4.5. Atomic Fluctuation

The atomic fluctuations were calculated for every atom and averaged for each residue, during the last 15 ns of MD simulations, using the cpptraj module available within the AMBER 12 package [24].

## Figures and Tables

**Table 1:** The binging free energy Δ*G* in kcal/mol of colchicine and paclitaxel with human and *Colchicum autumnale* tubulin.

Species	Ligand	Generalized Born Δ*G* (kcal/mol)	Poisson–Boltzman Δ*G* (kcal/mol)
Homo Sapiens	Paclitaxel	−23.25	−43.30
*C. autumnale*	Paclitaxel	−20.78	−44.81
Homo Sapiens	Colchicine	−51.79	−76.68
*C .autumnale*	Colchicine	−43.44	−64.71

**Table 2:** Binding free energy (kcal/mol) decomposition into key residues mediating the tubulin–ligand interaction.

Ligand	Species	Residue	Δ*E* vdw	Δ*E* elec	Δ*E* Polar	Δ*E* Non p	Δ*G* Total
Paclitaxel	Homo Sapiens	βLeu215	−2.90	0.48	0.43	−0.36	−2.36
		βThr274	−0.48	−2.91	2.07	−0.05	−1.37
		βSer275	−1.69	−2.00	1.77	−0.14	−2.07
		βArg276	−2.69	−4.00	4.75	−0.43	−2.28
		PTX	−18.28	−15.35	22.19	0.19	−14.40
Paclitaxel	*C. autumnale*	βLeu272	−2.10	0.19	0.72	−0.28	−1.84
		βThr274	−1.02	0.53	0.11	−0.11	−0.49
		βIle358	−2.28	−1.22	2.22	−0.50	−1.78
		βLeu360	−1.65	−0.24	0.46	−0.29	−1.72
		PTX	−18.23	−15.11	22.93	−3.36	−12.77
Colchicine	Homo Sapiens	αAla180	−1.53	−2.19	0.74	−0.14	−3.13
		αVal181	−0.89	−2.52	1.00	−0.055	−2.46
		βCys239	−0.80	−0.76	0.52	−0.10	−1.12
		βLeu246	−2.71	−1.08	1.00	−0.28	−3.07
		βAla248	−1.77	−0.26	0.48	−0.13	−1.67
		βLeu253	−2.68	−0.30	−0.089	−0.28	−3.36
		βAla314	−1.24	−0.32	−0.080	−0.18	−1.82
		βLys350	−2.24	−0.25	0.89	0.17	−1.77
		CLN	−32.12	−14.78	23.65	−4.96	−28.22
Colchicine	*C. autumnale*	αSer180	−1.43	−1.53	0.43	−0.19	−2.72
		αVal181	−1.19	−2.35	0.93	−0.058	−2.66
		βLeu246	−2.58	−0.55	0.75	−0.52	−2.90
		βLeu253	−2.25	−0.57	0.50	−0.22	−2.57
		βLeu257	−2.13	−0.21	0.20	−0.19	−2.33
		βLys350	−2.35	−2.20	1.46	−0.30	−3.38
		CLN	−27.14	−12.85	20.86	−4.42	−23.55

## Supplementary Materials

Supplementary materials can be found at www.mdpi.com/1422-0067/18/8/1676/s1.

## Abbreviations

APBS	Adaptive Poisson–Boltzmann Solver
BLAST	Basic Local Alignment Search Tools
CLN	Colchicine
GDP	Guanosine Diphosphate
GTP	Guanosine Triphosphate
MD	Molecular Dynamics
MM/PBSA	Molecular Mechanics Poisson–Boltzmann Surface Area
MM/GBSA	Molecular Mechanics Generalized-Born Surface Area
MT	Microtubule
RMSD	Root-Mean-Square Deviation
SASA	Solvent Accessible Surface Area

## References

[1] Hyams J.S., Lloyd C.W. (1994). Microtubules.

[2] Mitchison T., Kirschner M. (1984). Dynamic instability of microtubule growth. Nature.

[3] Jordan M.A., Wilson L. (2004). Microtubules as a target for anticancer drugs. Nat. Rev. Cancer.

[4] Wallace S.L. (1974). Colchicine. Colchicine.

[5] Weisenberg R.C., Borisy G.G., Taylor E.W. (1968). The colchicine-binding protein of mammalian brain and its relation to microtubules. Biochemistry.

[6] Ravelli R.B., Gigant B., Curmi P.A., Jourdain I., Lachkar S., Sobel A., Knossow M. (2004). Insight into tubulin regulation from a complex with colchicine and a stathmin-like domain. Nature.

[7] Luis L., Serrano M.L., Hidalgo M., Mendoza-Leon A. (2013). Comparative analyses of the β-tubulin gene and molecular modeling reveal molecular insight into the colchicine resistance in kinetoplastids organisms. BioMed Res. Int..

[8] Mane J.Y., Klobukowski M., Huzil J.T., Tuszynski J. (2008). Free energy calculations on the binding of colchicine and its derivatives with the α/β-tubulin isoforms. J. Chem. Inf. Model..

[9] Gigant B., Cormier A., Dorleans A., Ravelli R.B., Knossow M. (2009). Microtubule-destabilizing agents: Structural and mechanistic insights from the interaction of colchicine and vinblastine with tubulin. Top. Curr. Chem..

[10] Tuszynski J.A., Craddock T.J., Mane J.Y., Barakat K., Tseng C.Y., Gajewski M., Winter P., Alisaraie L., Patterson J., Carpenter E. (2012). Modeling the yew tree tubulin and a comparison of its interaction with paclitaxel to human tubulin. Pharm. Res..

[11] MOE-Molecular_Operating_Environment. https://www.chemcomp.com/announcements/2013-10-23-MOE2013.08.pdf.

[12] Matasci N., Hung L.H., Yan Z., Carpenter E.J., Wickett N.J., Mirarab S., Nguyen N., Warnow T., Ayyampalayam S., Barker M. (2014). Data access for the 1000 plants (1kp) project. GigaScience.

[13] Altschul S.F., Madden T.L., Schaffer A.A., Zhang J., Zhang Z., Miller W., Lipman D.J. (1997). Gapped blast and psi-blast: A new generation of protein database search programs. Nucleic Acids Res..

[14] Larkin M.A., Blackshields G., Brown N.P., Chenna R., McGettigan P.A., McWilliam H., Valentin F., Wallace I.M., Wilm A., Lopez R. (2007). Clustal w and clustal x version 2.0. Bioinformatics.

[15] Lowe J., Li H., Downing K.H., Nogales E. (2001). Refined structure of α β-tubulin at 3.5 a resolution. J. Mol. Biol..

[16] Krebs A., Goldie K.N., Hoenger A. (2005). Structural rearrangements in tubulin following microtubule formation. EMBO Rep..

[17] Kollman P.A., Massova I., Reyes C., Kuhn B., Huo S., Chong L., Lee M., Lee T., Duan Y., Wang W. (2000). Calculating structures and free energies of complex molecules: Combining molecular mechanics and continuum models. Acc. Chem. Res..

[18] Genheden S., Ryde U. (2015). The MM/PBSA and MM/GBSA methods to estimate ligand-binding affinities. Expert Opin. Drug Discov..

[19] Bhattacharyya B., Panda D., Gupta S., Banerjee M. (2008). Anti-mitotic activity of colchicine and the structural basis for its interaction with tubulin. Med. Res. Rev..

[20] Hastie S.B. (1991). Interactions of colchicine with tubulin. Pharmacol. Ther..

[21] Jolliffe I.T., Cadima J. (2016). Principal component analysis: A review and recent developments. Philos. Trans. A Math. Phys. Eng. Sci..

[22] UniProt C. (2013). Update on activities at the universal protein resource (uniprot) in 2013. Nucleic Acids Res..

[23] Nogales E., Wolf S.G., Downing K.H. (1998). Structure of the α β tubulin dimer by electron crystallography. Nature.

[24] Pearlman D.A., Case D.A., Caldwell J.W., Ross W.S., Cheatham T.E., DeBolt S., Ferguson D., Seibel G., Kollman P. (1995). Amber, a package of computer programs for applying molecular mechanics, normal mode analysis, molecular dynamics and free energy calculations to simulate the structural and energetic properties of molecules. Comput. Phys. Commun..

[25] Sondergaard C.R., Olsson M.H., Rostkowski M., Jensen J.H. (2011). Improved treatment of ligands and coupling effects in empirical calculation and rationalization of pka values. J. Chem. Theory Comput..

