# Supplementary material for: Modeling the Colchicum autumnale Tubulin and a Comparison of Its Interaction with Colchicine to Human Tubulin
Source: Int J Mol Sci. 2017 Aug 2;18(8):1676. doi: 10.3390/ijms18081676 (PMC5578066; doi:10.3390/ijms18081676)
Supplement: Supplementary file 1 [file ijms-18-01676-s001.pdf]

Modeling the *Colchicum autumnale* tubulin and a comparison of its interaction with colchicine to human tubulin

Ivana Spasevska, Ahmed T. Ayoub, Philip Winter, Jordane Preto, Gane K.-S. Wong, Charles Dumontet and Jack A. Tuszynski

Supporting information

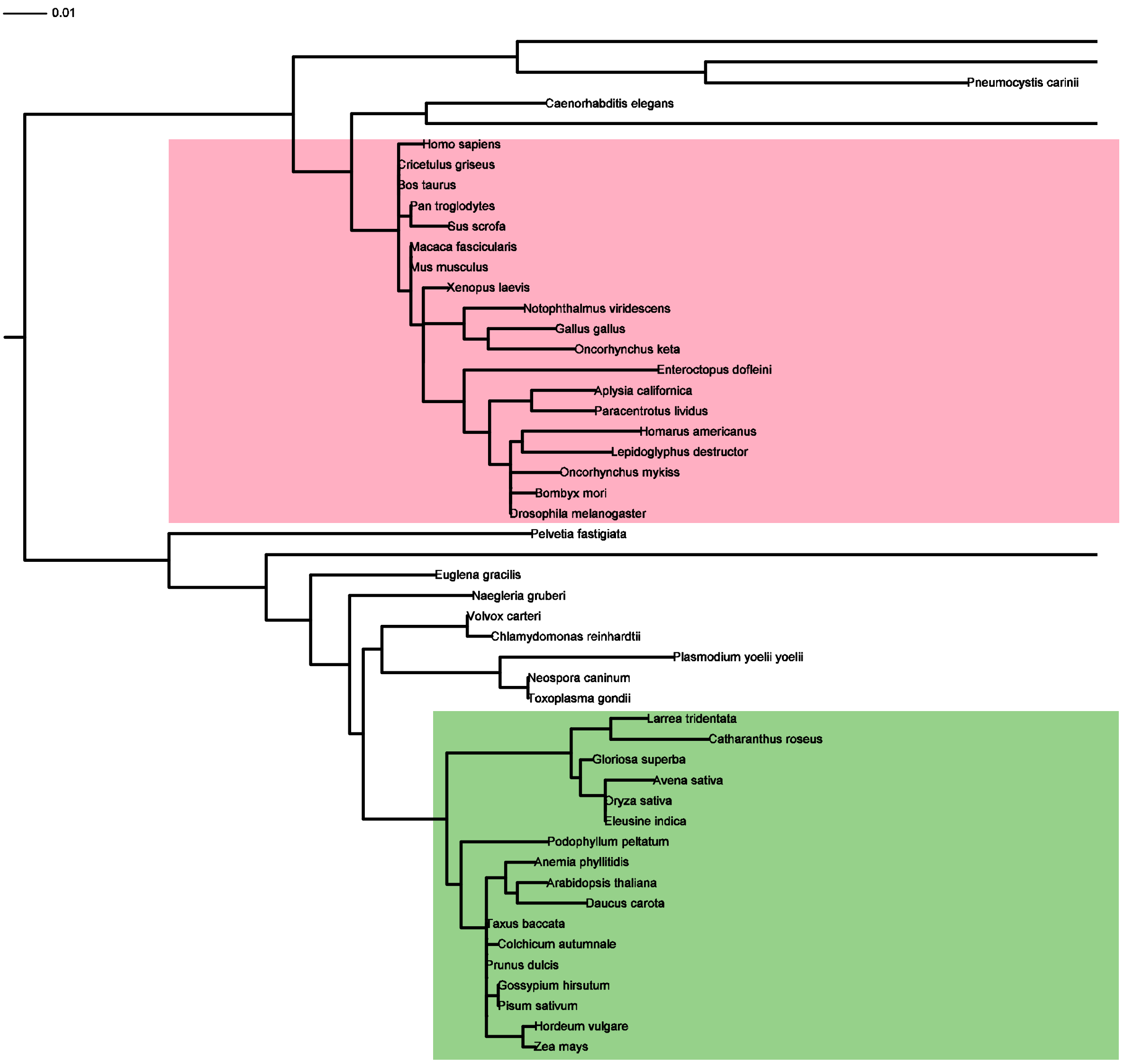

Figure S1. Phylogenetic tree of α-tubulin. Animals and plants groups are highlighted.

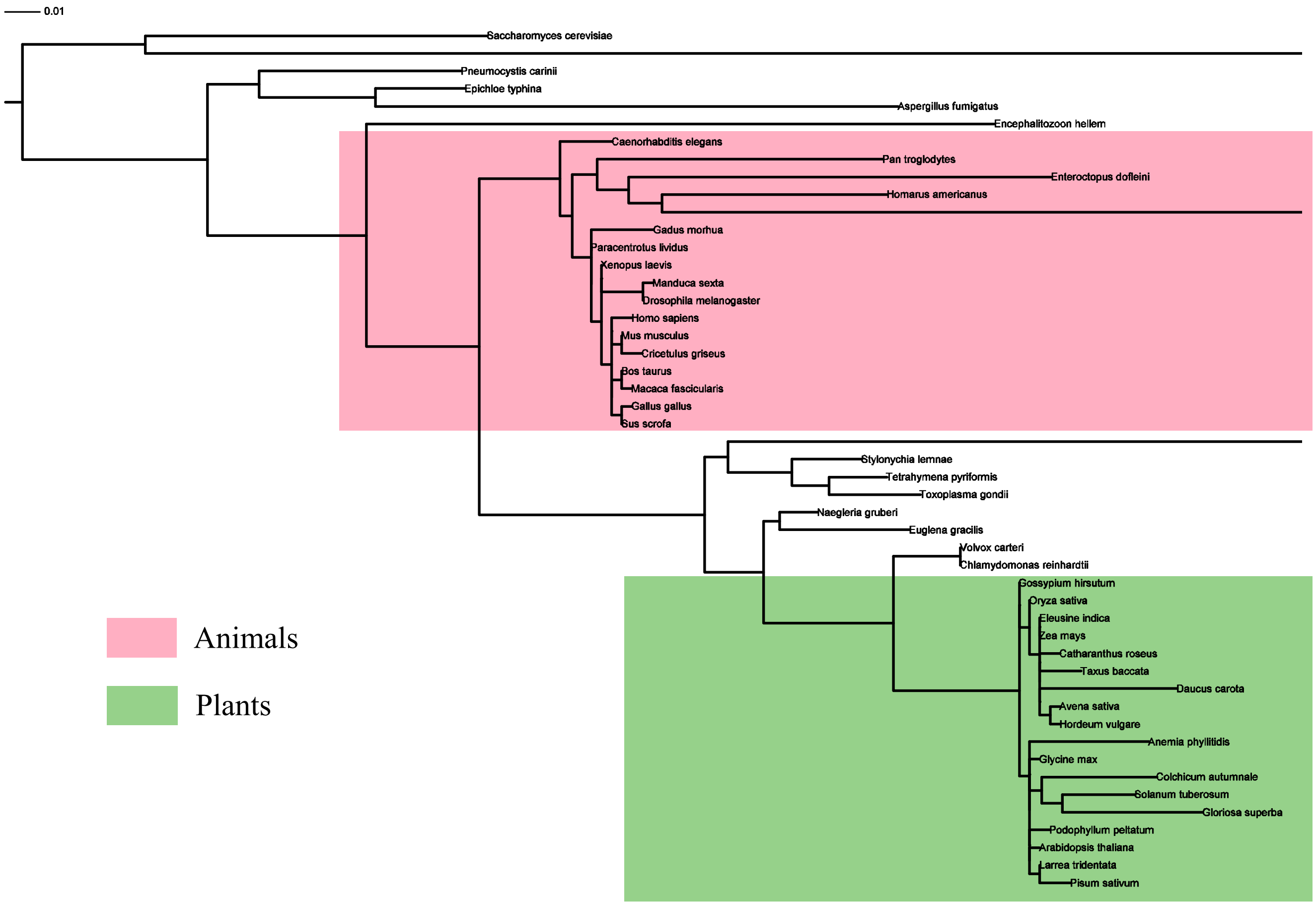

Figure S2. Phylogenetic tree of β-tubulin. Animals and plants groups are highlighted.

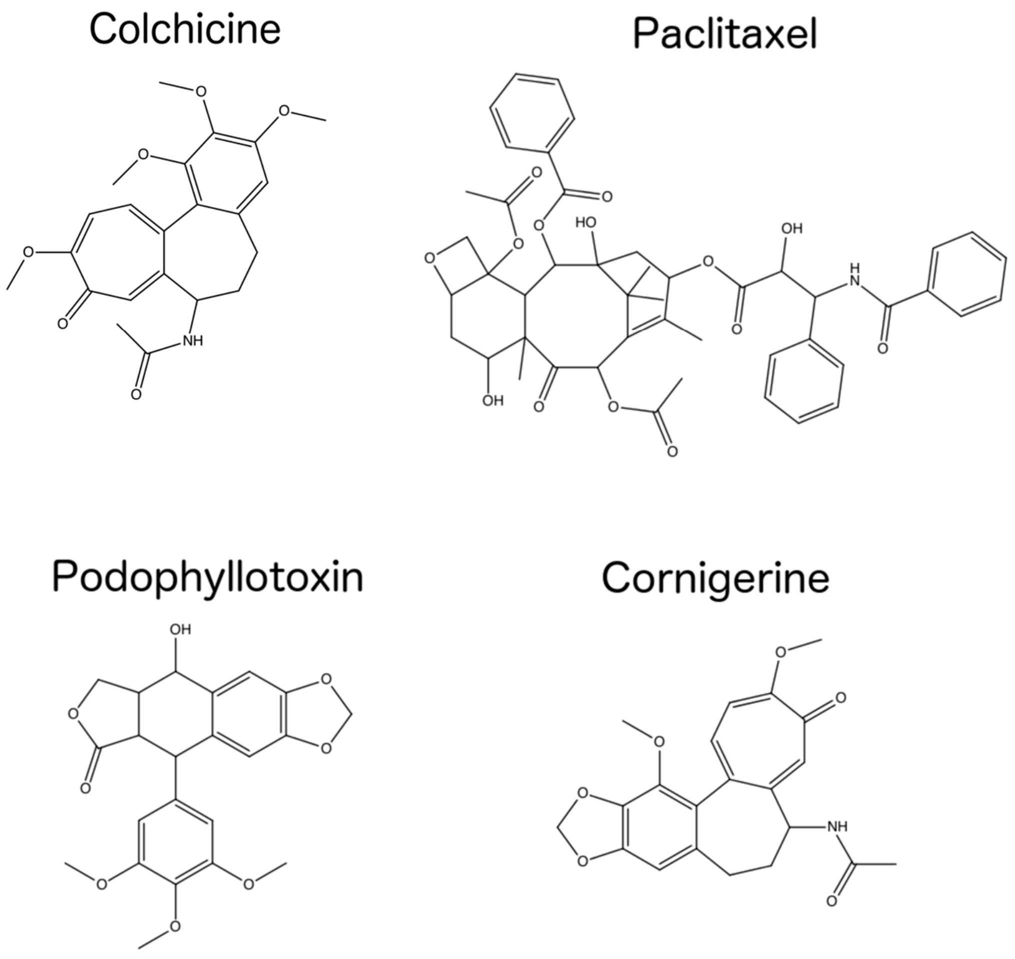

Figure S3. Comparison of colchicine and paclitaxel structures together with two other colchicine derivatives, i.e., podophyllotoxin and cornigerine.
